# Oligoantigenic Diet Improves Children’s ADHD Rating Scale Scores Reliably in Added Video-Rating

**DOI:** 10.3389/fpsyt.2020.00730

**Published:** 2020-08-20

**Authors:** Anna Dölp, Katja Schneider-Momm, Philip Heiser, Christina Clement, Reinhold Rauh, Hans-Willi Clement, Eberhard Schulz, Christian Fleischhaker

**Affiliations:** ^1^ Department of Child and Adolescent Psychiatry, Psychotherapy and Psychosomatics, Medical Center—University of Freiburg, Faculty of Medicine, University of Freiburg, Freiburg, Germany; ^2^ Clinic of Child and Adolescent Psychiatry, Psychotherapy, and Psychosomatics, SuedharzHospital Nordhausen, Nordhausen, Germany

**Keywords:** attention-deficit/hyperactivity disorder, children, adolescent, ADHD rating scale IV, brain-gut axis, oligoantigenic diet, inter-rater reliability, video rating

## Abstract

**Objectives:**

The influence of food intake on behavioural disorders was already described in the early 20th century. Elimination of individually allergenic food items from individual diets [“oligoantigenic diet” (OD)] showed promise to improve attention-deficit/hyperactivity disorder (ADHD) symptoms. However, only few of the positive results were evaluated by blinded symptom rating. Therefore the present study’s purpose was to evaluate the reliability of a non-blinded rating of the ADHD Rating Scale IV (ARS) for the assessment of OD effects in comparison to a blinded rating of the ARS based on pseudonymized video recordings.

**Methods:**

Ten children (8m/2f) aged 8 to 14 with ADHD according to ICD-10 participated in an uncontrolled, open-label dietary intervention study. Food items, commonly related to intolerances, were eliminated for four weeks. Participants with > 40% improvement in the ARS between T1 (before the diet) and T2 (after the diet) were defined as responders. Nutrients with individual relevance to ADHD symptoms were identified in a following reintroduction phase (T3–T4) lasting 8–16 weeks. The ARS was completed by a non-blinded child and adolescent psychiatrist (T0-T4). Sessions were recorded on video, pseudonymized, and evaluated by three blinded raters. Complete data were captured for eight children. The inter-rater reliability between the non-blinded therapist and every blinded rater was determined by the intra-class correlation coefficient (ICC). Correlations according to Pearson and Spearman between the non-blinded and blinded rating were calculated for each rater.

**Results:**

Two blinded raters and the non-blinded rater considered 5 of 8 (62.5%) children as responders, whereas one blinded rater disagreed as to the success of one case thus considering only 4 of 8 children as responders to the diet. Inter-rater reliability was assessed after each rater having scored 33 videos: The intra-class coefficients were >.9 for all raters (rater 1: *ICC*=.997, rater 2: *ICC*=.996, rater 3: *ICC*=.996) and the Spearman *rho* between the raters were high (*n*=33; rater 1: *rho* =.989, *p*<.0001, rater 2: *rho*=.987, *p*<.0001, rater 3: *rho*=.984, *p*<.0001), respectively.

**Discussion:**

As both, blinded and non-blinded ratings of the ARS, revealed relevant significant improvement of ADHD scores in children following an OD in this uncontrolled trial, Randomized controlled trials appear as highly desirable in order to replicate these improvements and to establish reliable and unbiased effect sizes thereby fostering further more objective confirmatory measurements.

## Introduction

With a worldwide prevalence of 5.3% among children and adolescents under the age of 18, ADHD ranks among the most common behavioural disorders (1). High heritability has been demonstrated in twin studies (2–4). While genetics play an important role in the development of ADHD, the mechanism of its development has not been conclusively elucidated yet. It can be assumed that the clinically highly variable appearance also exists in etiology and pathophysiology. The pathomechanisms of ADHD still largely defy elucidation (5) with various suspected genetic and non-genetic contributing factors (2).

Several studies have shown that nutrition is a strong moderator of symptoms in ADHD (6–13). Strong effects of the elimination diet or for food diet have been observed (6, 7, 10, 12–16).

Nevertheless this treatment approach has not remained uncriticized. One main point of criticism of the efficacy of the OD is the lack of sufficient blinded data. Sonuga-Barke et al. (9) emphazised that the evidence existing in open studies for positive treatment effects was not confirmed in blinded studies.

In such studies ADHD Rating Scale IV (ARS) has been used with its two subscales of inattention and hyperactivity-impulsivity as primary outcome and as assessment of treatment results (17). The ARS is widely used as primary outcome in many ADHD studies (18–24).

Since non-blinded parent ARS ratings could be strongly biased, a blinded video rating of the ARS by experts was chosen to obviate this influence and to improve the methodology in studies investigating the efficacy of OD in children with ADHD.

Video rating is a method which has already been successfully established in numerous other studies and also proven useful in the assessment of inter-rater reliability (25–30). An interview (25), activities of patients (29), or a medical test (30) had been recorded. Independent raters evaluated the videos by using questionnaires. Some of the studies utilizing video rating are briefly described in the following.

Aye et al. (31) investigated the reliability of the test of gross motor development (TGMD-2), a scale for assessing gross motor skills in children. The execution of the skills of the participating children was recorded on video. These videos were evaluated independently by three raters using the TGMD-2. The inter-rater reliability was determined. To describe the inter-rater reliability the following statistical values have been used: Intra-class correlation coefficient (ICC) according to Portney and Watkins (32) and to Ciccheti (33), Cronbach’s alpha as described in DeVellis (34) and Pearson´s and Spearman´s correlation coefficients following Chowdhury (35). ICCs in these studies ranged from .769 to .990 and were always rated very high (25, 31, 36–38).

To determine inter-rater reliability all these studies applied the usual psychometric methods of ICC, Pearson correlation and Spearman rank correlation.

The aim of the current study was to evaluate whether the primary outcome measure of ARS ratings would be reproduced by blinded raters by applying ICC, Pearson correlation and Spearman rank correlation.

To assess changes of behaviour in more detail, additionally the ADHD questionnaire of parents of the diagnostic system for mental disorders (DISYPS-II FBB-ADHD), the Child Behaviour Checklist (CBCL/4-18), and the abbreviated Connor´s rating scale (ACS) were applied as secondary outcome measures. These questionnaires are widely used in clinical examinations (39–46).

## Materials and Methods

The study was approved by the local ethics committee (application number 111/14) in accordance with the World Medical Association’s Declaration of Helsinki. Patients and parents gave written informed consent before participating in the study.

### Participants

Recruitment took place at the inpatient and outpatient units of the Department of Child and Adolescent Psychiatry, Psychotherapy, and Psychosomatics of the Medical Center (University of Freiburg).

Patients were informed about the study by experienced child and adolescent psychologists and physicians. Some parents also indicated that their children’s teachers informed them about the study. Others became aware of information material on the internet or through the local press.

Interested participants were instructed in detail about the procedure and objectives of the study in group meetings or individually. The external diagnosis was confirmed with the semistructured screening interview Kiddie-SADS-Present and Lifetime Version (K-SADS-PL) after consent had been obtained from the children and parents for participation in the study. In addition, the parents signed a declaration of consent for the recording of the pseudonymized videos as well as a release from confidentiality to external study centers. None of the interested participants were excluded due to the exclusion criteria.

All children (*n* = 10) completed the diet phase. 80% males and 20% females participated, which is consistent with the general prevalence of ADHD in the population. Further information about the study sample is depicted in **Table 1**.

**Table 1:** Participants’ characteristics.

No. Included	10
Age (means ± SD (range))	10.45 ± 2.13 (8-14)
Gender m/f	8/2
	
**Diagnoses**	**Number**
F90.0	10
	
**Comorbidities**	
Dyslexia F81.0	5
ODD	2
Enuresis F98.0	2
Suspected reactive attachment disorder of childhood F94.1	1
Expressiv speech disorder F 80.1	1
Suspected Asperger’s syndrom F 84.5	1
	
No. completed diet	10
Responder/Non-responder	7/3
(40% improvement)	

### Inclusion and Exclusion Criteria

According to the study protocol, children and adolescents aged 7 to 18 attending at least the 2nd grade of a general education school and carrying a confirmed ADHD diagnosis according to ICD-10 were included in the study. In addition, both the participating children or adolescents and their parents had to agree with the study procedure and sign the informed consent.

Children and adolescents were excluded from participating in the study, if the child had severe concomitant diseases or neurological or organic comorbidities not submittable to dietary interventions. A lack of compliance of parents and/or children or a lack of reading or writing skills of legal guardians and/or children led to the exclusion of the study as well as a parallel drug therapy of ADHD, a parallel participation in other studies, or a special diet already followed by the children (e.g., vegetarian, vegan).

### Outcomes

The primary outcome was measured using the ADHD rating scale IV (ARS), (17), as in the previous group of the study “Oligoantigenic diet in children and adolescents with ADHD” in Freiburg (47). The items of the ARS were translated into German (48, 49).

The ARS contains 18 items, 9 each on the inattention subscale and 9 on the hyperactivity/impulsivity subscale. The 18 questions can be answered with “Never or rarely” (= 0), “Sometimes” (= 1), “Often” (= 2) or “Very often” (= 3). For example Item 9 from the inattention subscale reads as follows: “Is forgetful in daily activities.” Thus, the ADHD Rating Scale is suited to determine the severity of ADHD symptoms by interviewing parents or teachers.

The ARS was filled out at every appointment by the study clinician by questioning the parents in the presence of the child. A symptom improvement of more than 40% between the two appointments T1 (before the diet) and T2 (after the diet) was defined as a response (7). As the study clinician spoke German to the participants and their parents, she used translated items of the ARS.

Other questionnaires for assessing behaviour, the Childhood Behavior Checklist (CBCL/4-18) (50) and the DISYPS-II FBB-ADHD (51) were filled in by the parents in the week before the next outpatient appointment. In addition, the ACS (52) was completed daily during the study period by both parents and the children’s teachers. Furthermore, a detailed nutrition record had to be kept over the course of the whole study, which also had to include abnormalities such as physical complaints. Therefore a daily nutrition- and health-diary was kept (53).

### Procedure

**Figure 1:**
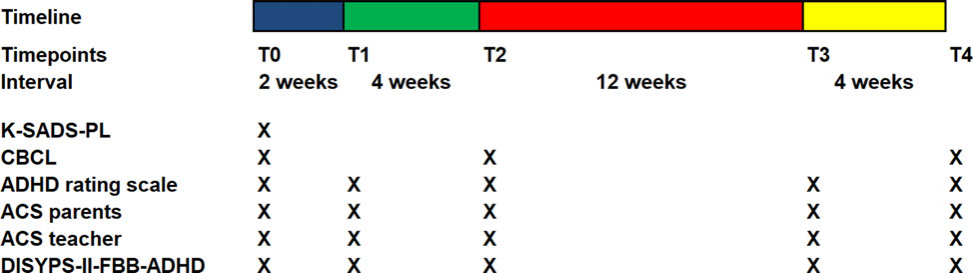
Timescales and measures for each appointment. Blue bar: pre-diet phase (2 weeks), normal eating. Green bar: diet phase (4 weeks). Red and yellow bar: reintroduction phase (6 + 10 weeks). T0: Baseline and beginning of documentation, T1: Start of OD, T2: Start of reintroduction phase, T3: 6 weeks after start of reintroduction, T4: after completion of reintroduction.

**Figure 1** gives an overview of the interventions at the different time points.

The first date of the study (T0) was defined as baseline. On this date, the diagnosis was checked with the help of the semistructured interview Kiddie-SADS PL. A medical history was taken with a focus on past and present symptoms and findings of allergies or food intolerances followed by a complete psychiatric interview and a standard neurological and medical examination, as carried out by the study clinician.

This was followed by four further outpatient appointments T1–T4. **Figure 1** depicts the timeline of acquistion of the questionnaires. The phases between the appointments will now be discussed briefly. Further study details are described by Blazinsky et al. (47) in this issue.

Phase T0-T1 extended over two weeks. During this time, the children were requested to eat as usual. The questionnaires had to be filled out and a food- and health-diary had to be completed. In addition, this period served as an intensive preparation phase for the diet.

During the following 4 weeks, between T1 and T2, children were only allowed to eat a limited selection of hypoallergenic foods. The structure of the diet was based on the study protocol of Egger and Pelsser (7, 12) (e.g., pig- and cowmeat, wheat- soy-, and cornproducts were not allowed, lamb- and turkey meat, potatoe-, rice-, and several vegetables were allowed). Supplementation of vitamins and minerals was advised. The families were supervised throughout the study by an experienced nutritionist in order to minimize the risk of malnutrition and to facilitate the implementation of the diet in the families.

All children “with an improvement of at least 40% on the ARS” according to Pelsser et al. (2011) between T1 and T2 were considered to be responders (7) to the diet. The non-responders could finish the study at this point and were transferred to treatment as usual. For the responders, an association of food intolerance and AHDH symptoms could be expected. Therefore, they started with the reintroduction phase after the four weeks of diet. During this period (T2–T4) different food groups were successively tested and individual reactions concerning the behaviour or physical complaints occurred. Individual food reactions led to the personal recommendation to avoid the consumption.

Approximately 6 weeks after the beginning of the reintroduction, a control appointment T3 took place. After testing all usually consumed nutrients, the last study appointement was T4. At this point a personal dietary recommendation was drawn up for each child, based on the previous testings. The children were asked to avoid the suspected foods for one year. Parents were informed that suspected foods could be retested after this period.

### Video

ARS surveys were recorded on video. The data up until T2 were important in order to compare the assignment into the two categories of responders or non-responders by the different raters.

The videos were recorded after the patient, the parents and the study clinician had signed the consent form. Recordings were accomplished with the camera NIKON Coolpix L830. The camera was installed in an upper corner of the room in a 4m by 4m room. The clinician, the mother and the child were sitting at a round table in the middle of the room so that they could be filmed face to face. In this way, the recording could be made inconspicuously in order to influence the situation as little as possible by the camera. Due to technical problems 4 videos could not be recorded.

After collecting all video recordings, they were edited with Wondershare Filmora software (version 8.5.0). All of the therapist’s evaluative statements as well as the personal information concerning the patients were deleted. Also, the prescribed diet was not allowed to be mentioned in the edited version of the video. Information about the appointment were removed if stated. In order to prevent the reading of the therapist’s hand movements as she wrote, the corresponding areas were pixelated in the video. The focus of the video should be on the participants and parents rather than on the therapist. A randomized list of the videos was created. Three raters were asked to watch the videos in the order determined by this list and to fill out the ARS during watching each video The raters are professional members of child and adolescent psychiatric hospitals experienced in the care of ADHD patients either as clinician, psychologist or nutritionist.

To transfer the files to the cooperating centers, the videos were saved in a container file encrypted by VeraCrypt (V.1.3.). The encrypted container files were copied to USB memory sticks and given to the external clinics. Opening instructions for the container files, as well as a blank version of the ARS, which was to be completed for each video were given to the external centers. The password to open the container file was sent separately to the external centers after the video files had been received. All files were deleted from the stick after evaluation.

### Statistical Analysis

The data of all ten participants were included in the statistical analyses. The primary assignment to responder/non-responder status was based on the threshold improvement in the ARS. Inter-rater reliability was determined based on the correlation and the degree of agreement of the overall ARS ratings between all raters for each video (*n* = 33).

IBM SPSS Statistics Version 24 was utilized for the statistical analyses.

We analysed complete datasets from before and after the diet. An ANOVA with repeated measurements was calculated for comparisons between the times of measurements.

In accordance to the studies mentioned in the introduction we computed Spearman rank correlations (*rho*) and Pearson correlations between the assessments of the ARS of the study clinician and the external raters. The *ICCs* (absolute agreement) were calculated to evaluate the inter-rater reliability using SPSS.

As for the descriptive statistics, means (*M*) and standard deviations (*SD*) were computed. Spearman correlation and estimated Cohen’s *d* according to the formula proposed by Morris & DeShon were calculated in order to report the effect size.

## Results

### Videos

All video recordings (*n* = 33) of the ARS questionaire were assessed by the internal clinician and the three external raters. Completed video data of the appointments T1 and T2 could be obtained for 8 children.

At each appointment T0 and T1 ten videos were taken. Further along the timeline, 8 videos were at T2, 4 videos at T3, and 1 video at T4.

The ICC was considered very high (33, 54) for all blinded raters. (*n* = 33, rater 1: *ICC* = .997, rater 2: *ICC* = .996, rater 3: *ICC* = .996). All *ICCs* were statistically significant (*p* <.001).

Even the lower limits of the confidence intervall for all raters were considered very high. Therfore, high inter-rater reliability can be assumed.

The number of available videos was not equal for each appointment. In order to test, whether this had an influence on the outcome, a more homogeneous sample with two videos of eight children was created. Therefore all ratings of the appointments T1 and T2 of the eight children having complete videodata were analysed. Lower limits and upper limits of the confidence intervall for all raters were considered very high as well. As the results in **Table 2** show, the *ICC* and Spearman correlations of the reduced sample (*n* = 16) are in agreement with the values including all videos (*n* = 33), which leads to the conclusion that the recording of a disparate number of videos at the appointments T0 to T4 does not influence the results.

**Table 2:** Pearson correlation coefficients among the parameters measured in the present study.

	Rater 1			Rater 2			Rater 3		
	Coefficient	[95% CI]	p	Coefficient	[95% CI]	p	Coefficient	[95% CI]	p
**ICC**									
*n*=33	.997	[.993, .998]	<.001	.996	[.991, .998]	<.001	.996	[.991, .998]	<.001
*n*=16^*^	.995	[.986, .998]	<.001	.994	[.983, .998]	<.001	.997	[.990, .999]	<.001
**Pearson *r***									
*n*=33	.994	[.988, .997]	<.001	.992	[.984, .996]	<.001	.992	[.984, .996]	<.001
*n*=16^*^	.990	[.971, .997]	<.001	.987	[.962, .996]	<.001	.994	[.982, .998]	<.001
**Spearman *rho***									
*n*=33	.989	[.978, .995]	<.001	.987	[.974, .994]	<.001	.984	[.968, .992]	<.001
*n*=16^*^	.995	[.968, .996]	<.001	.973	[.922, .991]	<.001	.984	[.953, .995]	<.001

* Videos of the 8 children with complete video data at appointments T1 and T2.

The correlations between the non-blinded rater and raters 1-3 were as follows: The Spearman correlation were *rho* = .989 (rater 1), *rho* = .987 (rater 2), and *rho* = .984 (rater 3) and significant for all raters (*p* <.001). The Pearson correlation were *r* = .994 (rater 1), *r* = .992 (rater 2) and *r* = .992 (rater 3) and also significant for all raters (*p* <.001).

**Figure 2:**
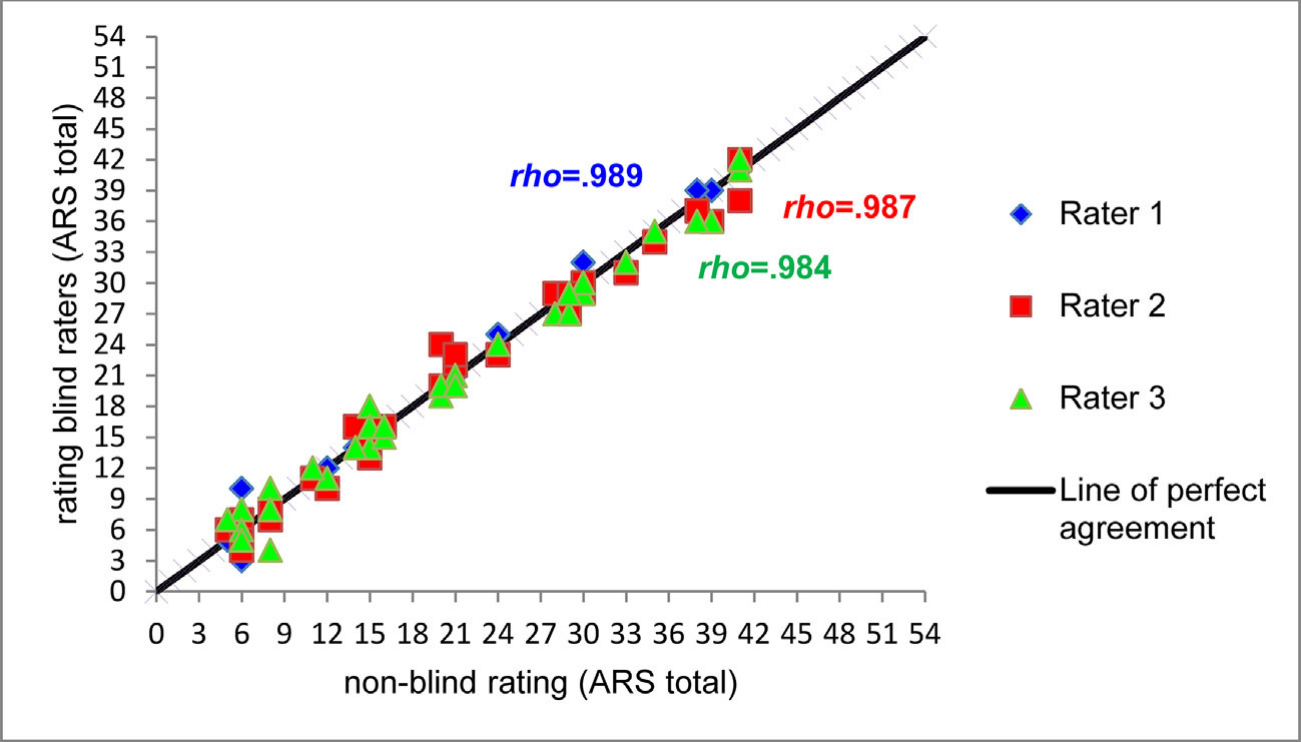
Scatterplot and Spearman correlations between non-blinded ARS total rating and three blinded raters (*n* = 33, Rater 1: *rho*(31) = .989, *p* < .001, Rater 2: *rho*(31) = .987, *p* < .001, Rater 3: *rho*(31) = .984, *p* < .001).

There were significant positive relationships between the ratings of the ARS (total) by the non-blinded rater and each of the the blinded external raters. According to the interpretation guidelines of Chowdhury et al. (35) this is a very strong positive correlation. For n = 16, Spearman *rho* and Pearson *r* were also extremely high, indicating that the results were not influenced by the unequal number of videos available for different appointments. As shown in **Figure 2**, the pairwise rating scores of the videos almost lie on the line of perfect agreement. Deviations between the ratings were independent from the absolute value of the ARS. Four videos were rated identically by all raters.

Some items of the ARS were answered very precisely by the parents, so there was not much room for interpretation by the raters. Of the 18 items of the ARS the parents answered 13.15 items on average by including the number or word of the scale into their answer (explicitly answered) and 4.85 questions by describing situations (not-explicitly answered). The proportion of total agreement among all raters expectedly was higher in the former (89%) than in the latter (72%).

### Primary Outcomes

Eight children had complete video data for the appointments T1 and T2. For two children no videos were available for one or both of the appointments T1 and T2. All videos were evaluated by each of the three external raters. As shown in **Table 3**, two of the external raters were completely consistent with the results of the study clinician. One external rater disagreed with the others as to whether one of the participants had reached the responder threshold.

**Table 3:** Assignment of the participants to responder/non-responder of the different raters [blinded (b) and non-blinded (nb)].

	Study clinician (nb)	Rater 1 (b)	Rater 2 (b)	Rater 3 (b)
Participant 1	Non-Responder	Non-Responder	Non-Responder	Non-Responder
Participant 2	Non-Responder	Non-Responder	Non-Responder	Non-Responder
Participant 3	Responder	Responder	Responder	Responder
Participant 4	Responder	Responder	Responder	Responder
Participant 5	Responder	No video	No video	No video
Participant 6	Responder	No video	No video	No video
Participant 7	Non-Responder	Non-Responder	Non-Responder	Non-Responder
Participant 8	Responder	Non-Responder	Responder	Responder
Participant 9	Responder	Responder	Responder	Responder
Participant 10	Responder	Responder	Responder	Responder

According to the assessment by rater 1, 50% of the children were to be considered responders. According to raters 2 and 3 there were 62.5% responders.

Between T0 and T1 ARS scores in the assessement by all raters. (*n* = 9, non-blinded rater: *p* = .272, rater 1: *p* = .307, rater 2: *p* = .546, rater 3: *p* = .432) were not significant.

The primary and secondary outcome measures are depicted in **Table 4**. Relying on the threshold of a 40% improvement on the ARS, by the study clinician (*n* = 10), about 60% of the children were responders.

**Table 4:** Results attention-deficit/hyperactivity disorder (ADHD) measures.

Measure	*n*	T1			T2			*df1*	*df2*	*F*	*p*	*r*	Estimated Cohen´s *d*
		*means*	*±*	*SD*	*means*	*±*	*SD*						
**ADHD Rating Scale (unblinded)**													
**total**	10	24.60	±	8.82	12.90	±	7.68	1	9	28.21	<.001	0.583	1.54
Inattention	10	12.70	±	4.72	6.20	±	3.55	1	9	15.18	0.004	0.210	1.23
Hyperactivity/Impulsivity	10	11.90	±	6.28	6.70	±	5.46	1	9	15.07	0.004	0.790	1.34
**ADHD Rating Scale total (all raters)**													
unblinded	8	23.38	±	9.38	13.63	±	8.26	1	7	20.84	0.003	0.773	1.61
blinded rater 1	8	23.50	±	9.52	14.00	±	8.35	1	7	17.19	0.004	0.744	1.47
blinded rater 2	8	23.63	±	9.75	13.75	±	8.28	1	7	24.5	0.002	0.816	1.75
blinded rater 3	8	23.25	±	9.63	13.75	±	7.03	1	7	16.2	0.005	0.721	1.42
**ACS**													
**total**	7	56.82	±	4.55	48.82	±	7.04	1	6	26.84	0.002	0.857	1.35
Restless-Impulsive	7	54.74	±	4.37	47.82	±	5.25	1	6	11.14	0.016	0.429	1.43
Emotional lability	7	60.02	±	9.41	53.00	±	8.71	1	6	27.91	0.002	0.429	0.77
**DISYPS-II FBB-ADHD**													
**total**	7	1.76	±	0.42	0.70	±	0.56	1	6	41.56	<.001	0.702	2.44
Inattention	7	1.93	±	0.43	0.92	±	0.45	1	6	33.34	0.001	0.439	2.18
Hyperactivity	7	1.35	±	0.85	0.51	±	0.72	1	6	10.38	0.018	0.631	1.22
Impulsivity	7	1.68	±	0.79	0.54	±	0.94	1	6	29.54	0.002	0.807	2.05
**Measure**	***n***		**T0**			**T2**		***df1***	***df2***	***F***	***p***	***r***	**Estimated Cohen´s *d***
**CBCL/4-18**													
**total**	10	68.60	±	5.19	60.40	±	5.50	1	9	40.45	<.001	0.711	2.01
Externalizing	10	67.00	±	5.14	60.40	±	4.35	1	9	27.92	0.001	0.665	1.67
Internalizing	10	63.70	±	9.02	58.10	±	5.72	1	9	10.6	0.010	0.818	1.03
Social retreat	10	61.70	±	8.59	56.90	±	6.14	1	9	6.04	0.036	0.695	0.78
Physical complaints	9	64.00	±	9.86	58.44	±	7.32	1	8	2.07	0.188	0.114	0.48
Anxious / Depressed	10	63.70	±	5.66	57.30	±	5.27	1	9	32.22	<.001	0.789	1.80
Social problems	9	63.44	±	7.33	58.11	±	5.99	1	8	3.21	0.111	0.113	0.60
Schizoid obsessive	10	58.00	±	8.83	51.80	±	3.79	1	9	4.35	0.067	0.060	0.66
Attention problems	10	68.00	±	9.87	63.70	±	8.56	1	9	1.44	0.260	0.244	0.38
Dissocial behaviour	10	62.20	±	7.10	55.50	±	3.54	1	9	15.78	0.003	0.686	1.26
Aggression	10	67.90	±	6.33	62.40	±	6.55	1	9	10.96	0.009	0.668	1.05

According to the ratings of the study clinician, including all 10 children, the mean improvement for the ARS scores after diet (T2 vs. T1) was significant (12.9 ± 7.68 vs. 24.6. ± 8.82 (mean ± SD); *F*: 28.21; *p* <.001). This was equally the case for the subscales “Inattention” (*p* = .004) and “Hyperactivity and Impulsivity” (*p* = .004) (see **Table 4**).

Including only the participants with complete video data on appointments T1 and T2 (*n* = 8), ARS score improved as rated by the study clinician (non-blinded rater) as well as by the external raters (see **Table 4**).

**Figure 3:**
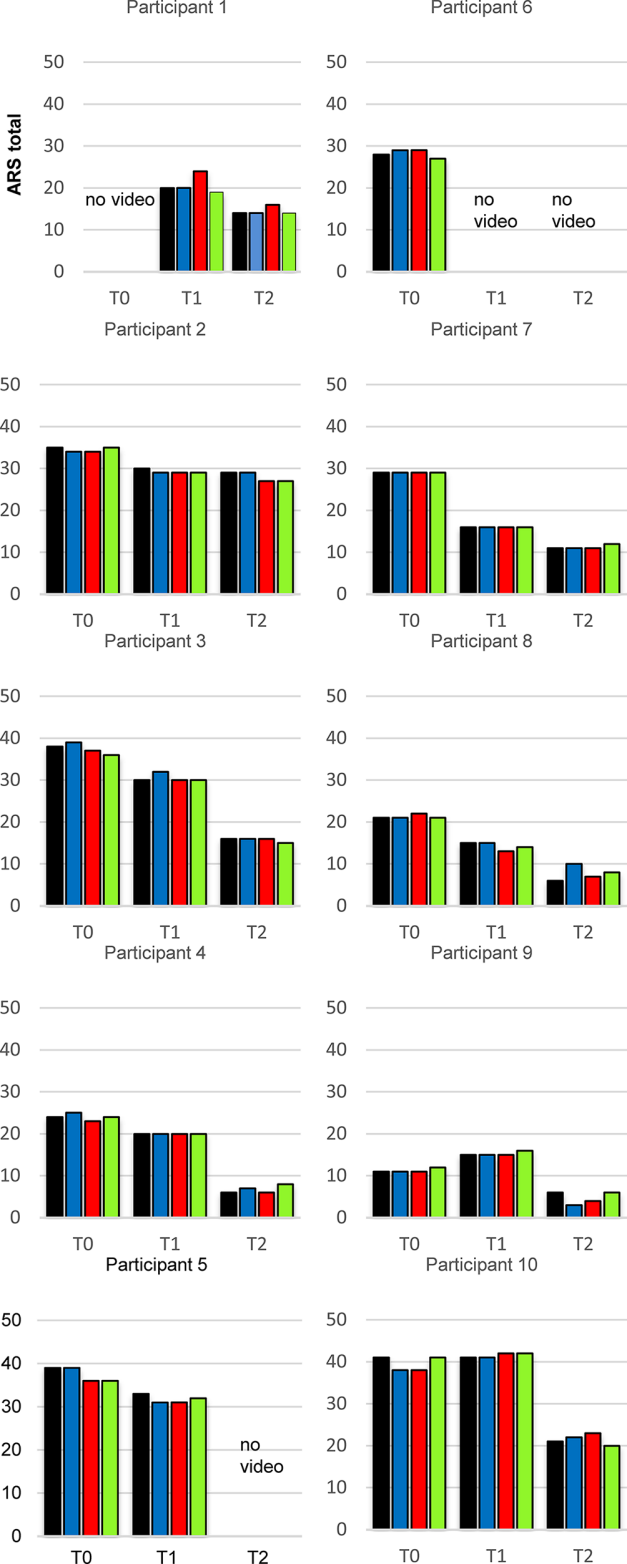
ADHD Rating Scale total data of every individual participant in the study at the appointments T0, T1 and T2 (n = 10), results for the 4 independent raters. Blue bar: non-blinded rater, red bar: video rater 1, green bar: video rater 2, yellow bar: video rater 3.

In **Figure 3** the ARS scores for all raters at the different time points are depicted for each child. Surprisingly participants 7 and 8 showed marked reductions of symptoms at T1 which cannot be explained by diet effects.

### Secondary Outcomes

To validate the results of the ARS, parents replied to the DISYPS-II FBB-ADHD questionnaire for one week retrospectively. The separate subscales for hyperactivity and impulsivity indicate that impulsivity is much stronger affected than hyperactivity. Further information could be detected in the subscales hyperactivity and impulsivity. The improvement after 4 weeks of OD in the DISYPS-II FBB-ADHD was statistically significant for every subscale (**Table 4**).

Changes of value in the CBCL/4-18 parent rating also showed statistically significant reductions 6 weeks after entering the study at T0. There was no assessment at T1 before the diet. Significant differences in the CBCL/4-18 were found for the subscales: “Externalizing” (*p* = .001), “Internalizing” (*p* = .01), “Withdrawn” (*p* = .036), “Anxious/depressed” (*p* <.001), “Delinquent behavior” (*p* = .003), “Aggressive behavior” (*p* = .009) (**Table 4**).

The values of the ACS dropped significantly after 4 weeks of OD in the ratings of the parents. The reduction was significant for both subscales “Restless-Impulsive” (RI) and “Emotional Lability” (EL) (**Table 4**). The compliance of the teachers was very low. Therefore, it was not possible to evaluate the ACS of the teachers.

## Discussion

Sonuga-Barke et al. (9) stated in their meta-analysis that OD led to significant symptom improvements in children with ADHD in unblinded studies, but that these results had yet to be proven on the basis of blinded data. They demanded, that “evidence of efficacy from blinded assessments is required before they are likely to be supported as ADHD treatments.” (9)

Similarly Pelsser et al. (6) and Rommelse & Buitelaar (55) recommended to repeat studies examining the effect of an OD in patients with ADHD with independent, blinded raters. This criticism and proposals motivated the design of the current study.

For the video rating, we found high ICCs. The inter-rater reliability can be classified as very good according to both definitions of Cicchetti (33) and Koo and Li (54).

The sample size and number of raters is important to ensure a high quality of the results. Koo and Li (54) recommended to include at least 30 subjects and 3 raters in reliability studies. To determine the ICC we used 33 videos rated by 3 independent blinded raters, which complies with this.

In this study, similar results in the blinded video rating of the ARS emerged between the scores by the blinded raters and those by the non-blinded raters. With a response rate between 50.0% (one blinded rater) and 62.5% (two external raters and the non-blinded rater), a similar proportion of responders as in previous studies was determined. The evaluation of a study in Freiburg (*n *= 24) showed a response rate of 64%, similar to the results of two studies by Pelsser et al. (6, 7) and Boris and Mandel (13) who found about 70% responders in their studies.

According to Storebrø et al. (20) a change of 6.6 points on the ARS is considered as the minimum of a clinically relevant difference, which was exceeded by the improvements between 9 and 27 points for the responders observed here between T1 and T2.

Thus, according to our present results both on the number of responders and on their achieved improvements, no marked deviations emerged between the initial non-blinded (47) and the subsequent video-aided blinded evaluations.

An advantage of this comparison taking place at the same site of Freiburg (apart of the subsequent videorating is the consistency of involved methods and personel (clinicians, nutrition).

Overall, no significant differences in ARS evaluation between T0 and T1 were observed, whereas significant differences between T1 and T2 were measured, which corroborates a positive effect of the diet on group level.

Looking at the individual probands, participant 7 stood out as far as the child showed a threefold relative improvement before the diet. A possible, not further investigated, explanation for this could be a rather common intolerance of inhaled food allergens (notably cooked legumes) which may have emanated from the table neighbours’ dishes at baseline, but not while the proband consumed his usual diet in the introductory test phase These effects might be modulated by the parasympathic system or/and involving emotion dysreguating domains (56–59). Contributing interactive or external study-related psycho-social factors and a more supportive attention from household partners might also explain this effect (55). Pelsser et al. (60), however, found no “signiﬁcant association between family structure and ADHD symptoms”.

In order to largely avoid disruptive factors in the current study, further interventions had to be avoided throughout. Exceptional circumstances had to be taken into account in the interpretation. Due to the long duration of the study, it was not possible to ensure completely identical family situations, making detailed documentation and individual consultation with the study supervisors even more important.

Ratings by the parents are important and used in ADHD of children both as means of diagnosis and monitoring of medication. Coghill et al. investigated the efficacy of Lisdexamphetamine in a six week placebo controlled trial and utilized inter alia a reduction of at least 30% reduction in the ARS as primary outcome measure. In a phase III study, which aimed “to establish the response to lisdexamfetamine dimesylate (LDX) in subgroups of patients with different ADHD medication histories” (61) the ARS was used to evaluate efficacy. Another study by Coghill et al. (62) “evaluate[d] the efficacy of LDX throughout the day” by using the Conners’ Parent Rating Scale (CPRS).

Some studies (8, 63) investigating the efficacy of OD resorted to a double blind placebo-controlled design. Kaplan et al. (8) found 42% responders, excluding placebo effects, in their study. They compared the behaviour of the participants in three phases. A baseline phase, (where the children ate as usual) with, a placebo-diet (concerning the same ingredients as in the baseline phase), and a restricted elimination diet. In the cross-over “placebo”-design by Schmidt et al. (63) there were 24% responders and therefore hidden severitiy factors like other facets of irritability might be suspected (64). 44% of the participants showed significant improvement in a second part of the study during methylphenidate therapy. This is remarkably lower than described elsewhere (70%–90%) (55, 65).

A weak point of the current study is the lack of blinding on the patient side. Furthermore, it has to be mentioned that though a very high inter-rater reliability and very large effect sizes in the ARS as well as in the DISYPS-II FBB-ADHD were determined, they have to be replicated with a bigger sample size. Although the sample consisting of 10 children is very small, the number of videos (*n* = 33) complies with the recommendation of Koo & Li (54). To further improve the study quality it would be better to investigate only one video per child.

It can be noted that the OD leads to a significant improvement of ADHD symptoms in the study participants. However, the sample certainly does not reflect the average patient with ADHD in the population. Instead, they are predominantly a subgroup of interested and motivated families. Some of them had mentioned prior to the study, that their kids’ behaviour seemed to be influenced by some of the foods eliminated in the oligoantigenic diet Nevertheless significant effects were also observed in an unselected group (63).

The blinded video-rating ensures an unbiased assessment of the raters. But it can still be criticized [see for example Rommelse & Buitelaar (55)] that the blinded rating is based on the non-blinded estimates of the parents. A classroom setting, as it was used in another study to compare the efficacy of different stimulant drugs in children with ADHD can actually be seen as the gold standard, providing “a controlled study environment” (66). The classroom setting was utilized in several other studies (66–70) and would include a behavioural observation (63, 71).

A number of reviews discussed the OD as a treatment option in children with ADHD (9, 14, 16, 72–78). Although the same studies were analysed, the authors of several reviews evaluate the effectiveness of the OD differently. Some authors described an unclear effectiveness of the OD (72, 74, 78). “Taken together it remains inconclusive whether elimination diets are effective as a treatment for children with [… ] ADHD” (78). Whereas others assessed the effectiveness to be relevant (16, 75, 77). These inconsistent evaluations emphasise the need of further studies on this subject (14, 74, 76). Stevenson (78) recommends a blinded rating.

Video-rating as well as other long available and certainly future digital technologies will hopefully boost trials also in the ADHD- and contiguous childhood psychiatric fields. Larger samples will allow for more robust results through OD including also mixed stimulant and OD arms, which increasingly may observe dopaminergic and other influences on the immune system (79). Similarily observations comparing hypoallergic diets among themselves (80) or with the “whole diet” (81) will allow the personalized shaping of proposable diets. This approach will join contiguous efforts oriented towards other “ill-defined gut-brain-axis-disorders” (82, 83), or biological investigations, e.g., of the microbiome (84), mast cells, even “unhealthy diets” (85), or fasting. Finally, the quite different, and under atomoxetine maybe less persistent, but also at times common and tragic subtype of concentration disorder with focus-wandering and “sluggish cognitive tempo” (86) may allow for further distinctions in nutritional, bacterial, and immune patterns also involving additional psychiatric (87) or neuro- behavioral dimensions like those involving sleep and taste (88).

## Conclusion

The current study confirms the assumption that an OD can lead to symptom reduction in children and adolescents with ADHD. Our findings largely matches findings in the literature that approximately 60% of patients show a significant improvement after 4 weeks of oligoantigenic diet and thus also confirm the hypothesis that food intolerances are a possible cause of ADHD.

In addition, a high inter-rater reliability could be demonstrated, which highlights the importance of the results from previous studies, even non-blinded ones. Double-blind placebo-controlled studies with a larger number of patients should be conducted to prove the efficacy and effectiveness of the OD.

## Data Availability Statement

The datasets generated for this study are available on request to the corresponding author.

## Ethics Statement

The studies involving human participants were reviewed and approved by Ethik-Kommission der Albert-Ludwigs-Universität Freiburg. Written informed consent to participate in this study was provided by the participants’ legal guardian/next of kin.

## Author Contributions

All the authors meet the following criteria: made substantial contributions to the conception or design of the work; or the acquisition, analysis, or interpretation of data for the work; and drafted the work or revised it critically for important intellectual content; and approved the final version to be published; and agreed to be accountable for all aspects of the work in ensuring that questions related to the accuracy or integrity of any part of the work are appropriately investigated and resolved.

## Conflict of Interest

The authors declare that the research was conducted in the absence of any commercial or financial relationships that could be construed as a potential conflict of interest.
